# New products

**DOI:** 10.1155/S1463924696000223

**Published:** 1996


Journal of Automatic Chemistry, Vol. 18, No. 5, (September-October 1996) pp. 191-192

### New products

### Centrifugal liquid extraction

The VectaSep CLE Extraction Device from Whatman International enables the new technique of centrifugal liquid extraction (CLE) to be used for aqueous sample preparation in the analytical laboratory.

VectaSep CLE offers many advantages when compared with conventional liquid-liquid extraction and other sample preparation techniques. It is designed and manufactured in a ready to use format, and requires just two dispensing steps (solvent and sample) prior to extraction. The remaining steps are performed in minutes. It is made from precision moulded, high purity polypropylene. It is a disposable sample extraction device, and has been created for use in 15 ml rotor cups within standard laboratory centrifuges. It comprises four parts: a tube with separate cap, a sample disperser with integral microporous membrane which forms the basis of the controlled centrifugal extraction process, and a separating cup.

Extraction is a simple two-step process. The first step involves the controlled extrusion of an aqueous sample from the sample disperser through the dispersion membrane into an extraction solvent by means of centrifugation. In the second stage, the sample disperser is replaced by a separating cup. A second, short centrifuge spin locates this cup at the solvent/sample junction, allowing analytes of interest to be selectively isolated.

VectaSep CLE offers numerous benefits compared with conventional procedures. Sample extracts are typically obtained within 30-60 min, thereby offering time savings of 50-80%, and significant cost savings per analysis. Yet at the same time, the accuracy, precision and linearity of data obtained is equal to or better than that generated by conventional liquid-liquid extraction.

VectaSep CLE is available in packs of 50 or 200 units, for use manually or automated with standard laboratory robots.

*For more information contact Whatman at Whatman House, St Leonard's Road, 20/20 Maidstone, Kent ME16 0LS, UK. Tel.: 01622 676670; fax: 01622 677011; E-mail HelenE@-Whatman.co.uk.*

### Gas chromatograph

*Enhancements for Outstanding HP 6890 Series GC System Performance* highlights several new capabilities of the HP 6890 gas chromatograph (GC) system that are designed to increase laboratory productivity:

(1) A new programmable temperature vaporizing (PTV) inlet, which allows large volume injections to improve trace analysis. It also introduces samples at low temperatures, reducing thermal degradation and inlet discrimination.
(2) A highly inert volatiles interface which is optimized for gas-phase sample-preparation systems such as purge-and-trap, headspace and air-toxic systems.
(3) Upgraded HP automatic liquid sampler which allows larger-volume injections, additional pre- and post-injection parameters and an eight-sample injector turret for greater flexibility and throughput.
(4) LAN capability.
(5) Open-interface module.

*Enquiries to Hewlett-Packard, Enquiry Fulfilment Department, PO Box 533, 2130 AM Hoofddorp, The Netherlands. Tel.: 4122 780 82 27; fax: 4122 780 83 21; Website http://www.hp.com/go/chem.*

### Diode array detector

The HP 1100 series diode array detector combines deuterium and tungsten lamps to provide high sensitivity over an extended wavelength range of 190-950 nm. Hewlett-Packard Europe's brochure on the detector gives an overview of ways in which to explore the spectral landscape for faster sample characterization. Full performance specifications are given as well as descriptions of the detector's built-in features that help labs with quality and regulatory compliance.

*Details from Hewlett-Packard (as before).*

### Demonstration disk for spectroscopy package

The GRAMS/32 demonstration disk covers the main components of GRAMS/32 including data importing, viewing, processing, organizing, and accessing. It features the data processing library with examples of the derivative, base-line correction, spectral subtraction, and smoothing applications as well as the 'macro wizard', a visual programming interface that allows users to customize their own data processing routines. The disk also demonstrates the data management functions of GRAMS/32's Spectral Notebase such as database creation, embedding and accessing OLE 2.0 compatible documents, and data searching and matching for spectra, chromatograms, text, and numeric entries.

*Copies from Galactic Industries Corporation, 395 Main Street, Salem, NH 03079, USA. Tel.: 603 898 7600; fax: 603 898 6228.*

### Atomic absorption

Perkin-Elmer's new AA spectrometers, the AAnalyst 100 and 300 Systems, offer automated wavelength and slit selection, double-beam, fully sealed optics and six-lamp motorized turret (optional for the AAnalyst 100). All configurations feature a large sample compartment,

0142-0453/96 $12·00 © 1996 Taylor & Francis Ltd

29 cm × 25 cm, redesigned for easy access, and a simplified quick-change burner system. Switching from flame to graphite furnace applications is simple using the new HGA™ 800 graphite furnace. A swing-arm design allows the furnace to swing into position, with no lifting of heavy furnace or autosampler components.

The AAnalyst 300 is a fully automated sequential multi-element instrument with built-in background corrector. All functions are controlled from a stand-alone PC running WinLab software under Windows 95. The AAnalyst 100 controls all parameters except gas flows from the keyboard and display panel. To meet changes in analytical requirements, with the addition of optional PC and Winlab software, full multi-element analysis can be performed.

*For further information, contact Perkin-Elmer Ltd, Post Office Lane, Beaconsfield, Bucks HP9 1QA, UK. Tel.: 01494 679269; fax; 01494 679389; E-mail: Greenca@perkin-elmer.com.*

### Laser sampler

The Model 330 Laser Sampler performs automatic solid sampling by laser ablation for direct analysis with ICP-MS or ICP-OES techniques. The high-performance excimer laser provides a pulsed laser beam focused down to 20 µm on the solid sample. Filled with a premixed KrF-gas mixture, the laser emits light of 248 nm wavelength only for optimum stability and intensity.

Laser sampling is versatile and can be used for a variety of difficult samples, including biological materials, geological thin sections, ferrous and nonferrous metals, semiconductor products, rare earth and uranium oxides.

The sample stage of the Model 330 is computer-controlled, using MS Windows-based software, providing precise X, Y and Z positioning. The changeover from laser sampling to standard liquid sampling is quick and simple on both ICP-OES and ICP-MS systems.

*For further information contact Perkin-Elmer Ltd (as before).*

### Sample preparation

A new technical note: *Sample Preparation for API LC-MS and CE-MS* (No. TN 200\LA Version 1) is available from Micromass Ltd. To ensure successful sample analysis by API LC-MS or CE-MS, there are suggested guidelines to keep in mind when preparing samples. Size and concentration, for example, are important. Although solid samples are preferable, this may not always be possible. The new literature gives details of the minimum sample concentration required when sending solutions to Micromass. The stated sizes are sufficient to allow optimization of the ionization of each particular sample before data are acquired, and the data are generated from much smaller sample sizes.

*Readers can log onto the Micromass home page via URL: http://www.micromass.co.uk. Or contact Micromass at Floats Road, Wythenshawe, Manchester M23 9LZ, UK. Tel.: 0161 945 4170; fax: 0161 998 8915.*

### Gas analyser

Balzers have announced a new Prisma gas analyser with mass range from 1 to 300 amu. Available from Pfeiffer Vacuum, this extended range makes the analyser ideal for monitoring many semiconductor fabrication processes, for example monitoring the tungsten CVD process. By analysing residual gases *in situ*, Prisma provides for the early recognition of potential vacuum system problems, such as leaks, saturation of cryopumps and contamination of process gases.

Although designed for monitoring multichamber processing systems in production, Prisma is versatile and is suited for R&D environments, such as high energy physics, and for general purpose gas analysis.

The analyser head and the electronics are incorporated in a single unit that is easily integrated into an existing vacuum system or process tool. Two filaments increase system running time, a new ion source design offers the highest sensitivity, and high power RF electronics ensures better mass selection as well as increased stability.

For fast interference-free data transfer over long distances, the Prisma gas analyser uses a LAN fibreoptic interface. It can also be used with Balzers' Quadstar software which runs under Windows. The Prisma can be easily controlled via a host computer.

*Further information from Pfeiffer Vacuum UK, Bradbourne Drive, Tilbrook, Milton Keynes MK7 8AZ, UK. Tel.: 01908 373333; fax: 01908 377776.*

### Plasma spectrometer

The recent IRIS/AP plasma spectrometer from Thermo Jarrell Ash has a CID solid state detector which observes the entire emission spectrum simultaneously; and Trace-Tech—axial plasma technology that provides excellent detection limit performance. The IRIS/AP also features Thermo Jarrell Ash's Echelle Optical System and ThermoSPEC CID Software.

*A brochure is available from Thermo Jarrell Ash, 27 Forge Parkway, Franklin, MA 02038, USA. Tel.: 508 520 1880; fax: 508 520 1732.*

